# Parasites as Drivers and Passengers of Human-Mediated Biological Invasions

**DOI:** 10.1007/s10393-015-1092-6

**Published:** 2016-01-28

**Authors:** Tim M. Blackburn, John G. Ewen

**Affiliations:** 1 Department of Genetics, Evolution and Environment, Centre for Biodiversity and Environment Research, University College London (UCL), Gower Street, London, WC1E 6BT UK; 2 Institute of Zoology, Zoological Society of London, Regent’s Park, London, NW1 4RY UK; 3 School of Earth & Environmental Sciences and the Environment Institute, University of Adelaide, Adelaide, SA 5005 Australia; 4 Distinguished Scientist Fellowship Program, King Saud University, P.O. Box 2455, Riyadh, 1145 Saudi Arabia; 5 Centre for Invasion Biology, Department of Botany and Zoology, Stellenbosch University, Stellenbosch, South Africa

**Keywords:** biotic resistance, enemy release, establishment, non-native species, novel weapons, spread

## Abstract

We provide an overview of the current state of knowledge of parasites in biological invasions by alien species. Parasites have frequently been invoked as drivers of invasions, but have received less attention as invasion passengers. The evidence to date that parasites drive invasions by hosts is weak: while there is abundant evidence that parasites have effects in the context of alien invasions, there is little evidence to suggest that parasites have differential effects on alien species that succeed versus fail in the invasion process. Particular case studies are suggestive but not yet informative about general effects. What evidence there is for parasites as aliens suggests that the same kind of factors determine their success as for non-parasites. Thus, availability is likely to be an important determinant of the probability of translocation. Establishment and spread are likely to depend on propagule pressure and on the environment being suitable (all necessary hosts and vectors are present); the likelihood of both of these dependencies being favourable will be affected by traits relating to parasite life history and demography. The added complication for the success of parasites as aliens is that often this will depend on the success of their hosts. We discuss how these conclusions help us to understand the likely effects of parasites on the success of establishing host populations (alien or native).

## Introduction

One of the primary ways in which humans are causing environmental change is by moving species to areas beyond the limits of their natural geographic distributions, where they may subsequently be introduced into the new environment and establish viable populations (Elton 1958; Williamson 1996; Lockwood et al. 2007; Blackburn et al. 2011a). These populations (and species) are here termed ‘aliens’. The first known example of an alien population dates from Australasia around 20,000 years BP, when fossil evidence suggests that people introduced a marsupial, the grey cuscus *Phalanger orientalis* (Diprotodontia, Phalangeridae), from New Britain to New Ireland (Grayson 2001). This introduction was the precursor to what is now a near ubiquitous global phenomenon. Species with alien populations currently number in the tens of thousands and derive from a wide range of taxa (Pimentel et al. 2001). Even so, the number of new alien populations and species continues to grow year on year (Genovesi et al. 2009; Roques et al. 2009; Blackburn et al. 2015a).

The ubiquity and diversity of aliens belies the fact that many introductions have failed to result in the establishment of alien populations (Williamson and Fitter 1996; Jeschke and Strayer 2005). Furthermore, those alien populations that have established have spread to greatly varying extents, with some expanding little beyond the site of introduction while others rank amongst the most widespread species in the recipient environment (Williamson et al. 2009). Some apparently well-established populations have subsequently declined, and even gone extinct; these collapses are occasionally dramatic (Simberloff and Gibbons 2004). Why some species establish as aliens while others fail, why some alien populations spread widely while others do not, and why some well-established alien populations collapse are core research questions in invasion biology (Lockwood et al. 2007; Blackburn et al. 2009; Davis 2009). Amongst the wide range of factors that have been argued to drive variation in the establishment success and extent of spread of alien populations are interactions with organisms (viral, bacterial, fungal, protozoan and metazoan) that are the causative agents of infectious disease: we hereafter refer to such organisms as parasites, and the species they infect as hosts. Parasites have been argued to affect the establishment and spread of alien host populations in three ways.

First, some alien host populations may escape the negative impacts on reproduction and survival they experience from parasites in their native geographic ranges. Alien host populations typically derive from very small numbers of introduced individuals (see, e.g. Blackburn et al. 2009), which may not be infected with many of their endemic parasites (Paterson et al. 1999; Prenter et al. 2004; MacLeod et al. 2010). If lower levels of parasite impact translate into increased population growth in the novel environment, then such alien host populations may be able to increase rapidly in numbers, escaping the stochastic effects that afflict small populations (Allendorf et al. 2013) and increasing the probability that they will be able to establish and spread. This is termed the Enemy Release Hypothesis (ERH; Keane and Crawley 2002).

Second, some alien host populations may benefit from the co-introduction of their parasites. If those parasites subsequently infect and cause population declines in native species that otherwise would have competed with or predated upon the alien host species, the alien host population may be more likely to establish and spread as a result. This idea was discussed by Price et al. (1986), and is known as the Novel Weapon Hypothesis (NWH).

Third, some alien host populations may suffer from increased negative impacts on reproduction and survival from novel parasites they encounter in their alien geographic ranges (Elton 1958). If these higher levels of parasite impact translate into decreased population growth rates in the novel environment, then such alien host populations may be less likely to escape the stochastic effects that afflict small populations, and hence to establish and spread. This is one aspect of what has been termed the Biotic Resistance Hypothesis (BRH; Lockwood et al. 2007).

There are at least two added dimensions to the issue of parasites as drivers of invasion success (or failure). If host species succeed or fail to establish alien populations (or to spread) because of the parasites carried by translocated individuals (or indeed if those parasites are neutral with respect to success), then the potential of parasites of those hosts to become alien species is also affected. Understanding the effects of the parasites of alien hosts on the likelihood that their hosts survive to establish and spread in novel environments also informs about the likelihood that those parasites will become alien species themselves. All of this is relevant because sometimes it is desirable that translocated host (or indeed parasite) species succeed in establishing viable populations, for example, because they are economically valuable species such as biocontrol agents, or because they are being introduced for the purposes of conservation, for example, through a process of assisted colonisation or ecological replacement (Seddon et al. 2014).

In view of the potential for parasites to be both drivers of, and passengers on, the success of translocated populations, here we present a review of the current state of knowledge of parasites in biological invasions. The aims of this paper are threefold. First, we will review what we know about the effects of parasites on the likelihood that their hosts will establish and spread when introduced to novel environments. Second, we will review what we know about the causes of the success of the parasites themselves as aliens. Finally, we will discuss how the conclusions from the first two aims help us to understand the likely effects of parasites on the likely success of populations we would like to succeed in establishing viable populations, whether those populations are alien or native.

## Parasites as Invasion Drivers

### The Enemy Release Hypothesis

There is evidence that host species that successfully establish alien populations, and that subsequently go on to spread across the new environment, tend to have escaped from parasites that afflict them in their native range (Mitchell and Power 2003; Torchin et al. 2003; Lymbery et al. 2010; Roche et al. 2010; Prior and Hellmann 2015). For example, Torchin et al. (2003) showed that parasite species richness and prevalence was generally lower in the alien than the native range for a variety of alien species, including molluscs, crustaceans, fishes, amphibians, reptiles, mammals and birds. Mitchell and Power (2003) showed that plant species introduced to the USA were infected by, on average, 84% fewer fungi and 24% fewer virus species in their alien versus their native ranges. Successful alien species may also tend to harbour fewer parasites than native species in the same community (Roche et al. 2010). For example, in Northern Ireland, the invading alien amphipod *Gammarus pulex* has lower parasite diversity than the native *G. duebeni celticus*, and also lower prevalence and burden of the two parasite species that the alien and native amphipods share (Dunn 2009). These patterns are concordant with release from parasites as a determinant of success in alien invasions.

However, there is as yet little convincing evidence that release from parasites is actually a determinant of success in alien host population establishment or spread. There are at least two reasons for this. First, it is difficult to demonstrate for any given alien host population that its success was due to enemy release and not to other factors. It is necessary to show that the native host population is controlled by enemies, that the alien host population has escaped this control, and that this escape is the key determinant of success (Prior et al. 2015). There are examples where release from enemies has occurred but does not appear to underlie success (e.g. McDonald and Kotanen 2010; Prior et al. 2014). Second, the mechanisms that lead to escape from parasites should apply to all alien host populations—successful or not (see below). Under the ERH, it is necessary for alien host populations that successfully establish to have benefitted more from parasite release than those that fail to establish, and likewise for those species that have versus have not spread (Blackburn et al. 2015a). Studies are only informative on these questions if they have compared the extent of escape from parasitism in host populations that are introduced and become established versus those that are introduced but do not, or in host populations that establish and spread to varying extents (van Kleunen et al. 2010). We are aware of only two studies that have adopted this approach. Mitchell and Power (2003) showed that, amongst plant species listed as natural area invaders, species that experienced more complete pathogen release were more widely invasive. However, their measure of invasion is not a direct measure of extent of spread, and their analysis is not robust to the exclusion of a single outlier. Van Kleunen and Fischer (2009) showed that the geographic spread of alien plants introduced from North America to Europe was negatively associated with their release from fungal pathogens, contrary to the ERH. Neither of these studies explores the extent to which the species were under enemy regulation in their native ranges.

There is abundant evidence that natural enemies regulate natural populations of animals and plants (Sih et al. 1985; Prior et al. 2015), and successes in the biocontrol of aliens demonstrate that reacquainting hosts with their parasites can have dramatic impacts on populations of the former (Lafferty et al. 2005). Hence, it might be considered surprising that there is so little direct support for an effect of escape from parasites on alien host invasion success. The reason is undoubtedly due in part to the absence of information with which to test the influence of parasites on establishment success: there are simply no data on the parasite loads of failed introductions with which to compare successes (van Kleunen et al. 2010). However, even if there were, we might be unlikely to identify an effect. Translocated host populations may lose parasites for three reasons. First, introduced populations tend to consist of relatively small numbers of translocated individuals (Blackburn et al. 2009). These individuals may by chance lack parasites due to sampling effects (Paterson et al. 1999; Prenter et al. 2004; MacLeod et al. 2010). Second, parasites may be lost as a result of reduced opportunities for transmission in the alien environment, for example, because host populations are typically small and host densities low in the early stages of an invasion (Dunn 2009). Third, translocated individuals with parasites (or the parasites themselves) may consistently die in transit (Prenter et al. 2004). This may especially be the case for highly virulent parasites (Strauss et al. 2012). Parasites lost in this way may be unable to reach new environments (Prenter et al. 2004), perhaps unless transit times are greatly reduced. This third reason would apply to all translocated hosts, meaning that successful and failed introductions could not be distinguished on the basis of parasite loss in this way.

The first two of the reasons why alien hosts might lose parasites would be expected to lead to higher likelihoods of loss, and hence higher establishment success under the ERH, when fewer individuals were translocated (Drake 2003). However, there is in fact a robust and consistent positive relationship between establishment success (and indeed extent of spread) and the number of individuals introduced (“propagule pressure”) for alien populations (Lockwood et al. 2005; Colautti et al. 2006; Hayes and Barry 2008; Simberloff 2009; Blackburn et al. 2015a, b). This latter relationship probably arises because larger propagule pressure buffers against the stochastic processes (demographic, environmental, genetic or Allee) to which small, introduced populations will be vulnerable (Duncan et al. 2014; Blackburn et al. 2015b). These effects seem to outweigh any benefits that smaller introduced populations may accrue by escaping parasites (Drake 2003; Dunn 2009). Drake (2003) suggests that enemy release may mediate variation in the subsequent extent of spread, but there is currently no good evidence that it does. It is also hard to see how it would, given that propagule pressure is also positively related to spread (Blackburn et al. 2015b).

**Fig. 1:**
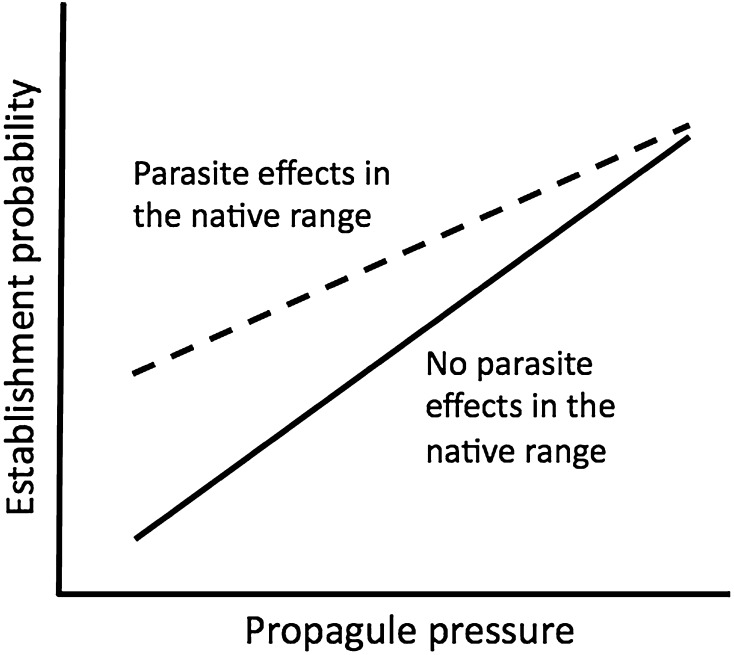
Hypothetical relationships between establishment success and propagule pressure (number of individuals introduced) for species for which native populations are (*dashed line*) or are not (*solid line*) regulated by parasites. Establishment probability increases with propagule pressure because of the effects of stochastic processes on small populations. However, for a given propagule pressure, success is higher for species more heavily impacted by parasites in their native range, because these species have more to gain from escape from these parasites. The lines converge because species are less likely to escape from their parasites as propagule pressure increases. Note, however, that escape from greater parasite impacts in the native range may also *decrease* the likelihood of success for a given propagule pressure, if those impacts are greater on the competitors of the introduced species, and therefore actually benefit it through apparent competition (Prior et al. 2015).

Positive relationships between success and propagule pressure suggest that escape from parasites is unlikely to be a primary driver of host invasion success, but it could still mediate variation around the propagule pressure relationship. Species vary in the extent to which their native populations are regulated by natural enemies, and so enemy release may matter more for the success of host species for which enemy impacts are naturally greater (Prior et al. 2015). For a given propagule pressure, host success may therefore be higher for species with more to gain by escaping their enemies (Figure 1).

### The Novel Weapons Hypothesis

Escape from parasites seems unlikely to distinguish successful from unsuccessful invasions, but an alternative idea is that success is greater for host species that bring their parasites with them. Theory predicts that the virulence of parasites will be low in hosts that have evolved with the parasite, but high in new hosts because of lack of evolved immunological resistance (Schmid-Hempel 2011). The parasites arriving in alien hosts are also expected to be relatively benign to those hosts, because otherwise the hosts are likely to have died in transit (Strauss et al. 2012). Not all parasites are equally likely to make the jump into new hosts, with generalist and vector-borne parasites being the most likely (Prenter et al. 2004; Hatcher et al. 2012). Nevertheless, if alien parasites can infect native species in the recipient environment (i.e. if there is parasite ‘spillover’; Daszak et al. 2000), if they are indeed more virulent in these naïve hosts, and if their negative impacts on the native species increase the likelihood that the alien host establishes and spreads, then these parasites may be considered to be novel weapons in the struggle between alien and native hosts. The NWH could also operate via ‘spillback’ (Daszak et al. 2000; Kelly et al. 2009). Kelly et al. (2009) argue that in some cases a native parasite may actually be less virulent in an alien host and facilitate invasion through spillback from alien to native host (see also Strauss et al. 2012).

There are a number of high-profile examples of host invasions that are likely to have been facilitated by parasite spillover to native competitors, usually close phylogenetic relatives of the alien species (Strauss et al. 2012). The classic example is the replacement of the red squirrel (*Sciurus vulgaris*) by the grey (*S. carolinensis*) in the UK, which has been mediated at least in part by parapoxvirus introduced from North America along with the grey squirrels (Sainsbury and Gurnell 1995; Tompkins et al. 2003; Bosch and Lurz 2012). The virus is highly virulent in red but not grey squirrels, and the greys act as a reservoir for it. While the grey squirrel is also a superior competitor, features of the invasion, such as the disappearance of red squirrels from areas before grey squirrels arrive, suggest that the virus is facilitating the invasion. Other examples of parasite-mediated invasions include the red signal crayfish (*Pacifastacus leniusculus*) in the UK and the harlequin ladybird (*Harmonia axyridis*) in Europe and North America (Strauss et al. 2012; Vilcinskas 2015). Parasites have also been implicated in plant invasions, although here the mechanism is spillback; spillover may be less common in plants because most parasites cannot accompany those species introduced as seeds (Mitchell and Power 2003; Strauss et al. 2012).

As with the ERH (albeit in reverse), it is not enough to show that alien hosts are accompanied by parasites to provide a valid test of the NWH: rather, variation in success must be linked to variation in parasite impacts. As with the ERH, there are simply no data on the parasites of species that failed to establish to compare against those that succeeded, and therefore, the validity of the NWH for the establishment stage of invasion is currently untestable. However, in this case, the robust positive relationship between establishment success (and extent of spread) and numbers of individuals introduced is at least consistent with the idea that success is higher for species that are more likely to bring parasites with them. There is more promise in testing the NWH for variation in the extent of alien spread, and the negative relationship between the geographic range size of alien plants and their release from fungal pathogen load found by van Kleunen and Fischer (2009) is also consistent with the hypothesis. Further tests in this vein would be a useful start in evaluating the potential generality of the NWH, on the assumption that alien species harbouring more alien parasites are more likely to be wielding a novel weapon.

Stronger evidence for the importance of novel weapons would come from demonstrations that more successful alien hosts are more likely to have reduced populations of their native natural enemies through parasite spillover (or spillback). However, any such test would need to overcome considerable hurdles. The effects of parasites can be difficult to detect, even in well-studied invasions. For example, there was a gap of more than 60 years between the first identification of the squirrel pox disease and the first suggestion that it might have a role in the decline of the red squirrel in the UK (Strauss et al. 2012). Parapoxvirus kills red squirrels very quickly, and so is rarely seen in the wild. Inconspicuousness is likely to be a feature of the kinds of highly virulent parasites that are most likely to regulate host populations (Anderson and May 1981). Furthermore, while there have undoubtedly been obvious and catastrophic population declines as a result of alien parasites (e.g. chestnut blight in North America, rinderpest in Africa, the chytrid fungus worldwide), most effects are likely to relate to less virulent but nonetheless persistent and important sub-lethal infections (Prenter et al. 2004). Effects of such parasites will be even harder to demonstrate. Finally, it is not enough to show that native and alien host species share parasites—one must demonstrate that the parasite has deleterious impacts on the native species, and improves the performance of the alien as a result (Strauss et al. 2012). Perhaps the best we can hope for is evidence that novel weapons matter for some invasions, but not how often they matter.

### The Biotic Resistance Hypothesis

The theoretical expectation that parasite virulence will be lower for co-evolved than for novel, naïve hosts (Schmid-Hempel 2011) may explain why novel weapons work, but should apply equally to alien host species encountering novel parasites endemic to the new environment. Indeed, arguably novel weapons should work more strongly *against* aliens. Alien species tend to be introduced in low numbers and therefore likely missing many of their natural parasites. Alien species are introduced into environments relatively species rich and these communities will tend to have relatively greater parasite species richness (Krasnov et al. 2004; Thieltges et al. 2011). In general, therefore, and acknowledging well-known counter-examples (e.g. avian malaria on Hawaii; Warner 1968), one would expect alien species to encounter more novel parasites than they bring. If novel weapons matter, we would expect alien host species to establish and spread less well in native assemblages with higher parasite diversity, as predicted by the BRH.

Biotic resistance can of course derive from elements of the native biota other than parasites, such as predators or competitors. Most tests of the BRH have addressed general relationships between establishment success or extent of alien host spread and indirect correlates of community richness, such as latitude or island versus continental location, or direct measures of community richness other than that of parasites (Blackburn et al. 2011b). These tests are far from convincing in their support for the BRH (Sol 2000; Blackburn et al. 2011b). As far as we are aware, there are as yet no studies that have explicitly tested whether native parasites are responsible for biotic resistance to aliens, although such negative relationships between correlates of native richness and alien success as do exist may be down to parasites. Once again, we would note the difficulty of demonstrating direct effects of parasites on the failure of alien host populations to establish, as failures typically disappear without study; the effects of biotic resistance may be greatly underestimated. Comparative analyses of relative levels of alien host success (establishment or spread) in areas with different parasite assemblages would be possible, though.

There are also few clear examples where the spread of alien host species into new areas have been prevented by native parasites. One classic example from agriculture relates to the impact of the protozoan parasite *Trypanosoma brucei* on cattle, which has acted as a major constraint to livestock production in parts of Africa (Perkins et al. 2008). The recruitment of native diseases may be responsible for at least some of the sudden and unexplained population crashes observed in some alien populations (Simberloff and Gibbons 2004); the rate of enemy accumulation seems to be driven by the extent of the alien host distribution rather than residence time (Strong et al. 1977; Branco et al. 2015). However, as noted above, the effects of parasites can be difficult to detect even in well-studied populations, and attributing an alien host population crash to native parasites would require extreme serendipity in terms of the type and timing of research on the alien.

One argument against a general effect of biotic resistance by parasites comes from tests of Darwin’s observation that it should be easier for alien host species with no close phylogenetic relatives to invade new areas, because they will tend to share fewer natural enemies with the native species (Darwin 1859). This idea has become known as Darwin’s Naturalisation Hypothesis, although Darwin did also recognise that the reverse could be true if shared environmental preferences mattered more than shared natural enemies (Diez et al. 2008). However, tests of the hypothesis have been equivocal in their support for it (Thuiller et al. 2010). If biotic resistance (of any kind) does affect the success of alien species, its signature has not yet been detected in patterns of relatedness between aliens and natives.

## Parasites as Invasion Passengers

Parasites (or the lack of them) can potentially affect the invasion success of their hosts, but parasites can themselves be alien species. Indeed, they may constitute a considerable proportion of all alien species. Given that around 40% of known animal species are parasites, that many protozoa, fungi, bacteria and plants are also parasitic (Dobson et al. 2008), and that the diversity of parasites is likely to be less well characterised than that of their hosts (Dobson et al. 2008), it is probably reasonable to suppose that around half of all species are parasites. Therefore, any assessment of the drivers of alien invasion success that does not consider the success of alien parasites may be covering only half the story.

Invasions by alien parasites can be considered using the same multi-stage framework as their hosts (Blackburn et al. 2011a; Lymbery et al. 2014): to become an invasive alien, a parasite species must be transported beyond the limits of its native distribution, be introduced into a new environment, establish a viable population there, and then subsequently spread. However, the challenges faced by parasites differ in several respects from those faced by their hosts. Insights into these challenges can also be gained from studies of disease emergence in novel hosts, and there is conceptual similarity between multi-stage models of the invasion process and of disease emergence—involving contact between the reservoir and novel hosts (= transport), spillover into the novel host (introduction), persistence in the novel host (establishment) and pandemic spread (spread) (Hatcher et al. 2012; Jeschke et al. 2013). While alien invasion by a parasite does not require transfer into a novel host, some of the processes influencing invasion into novel hosts and novel locations may be similar.

### Transport and Introduction

Invasions begin with individuals being transported to locations outside their natural geographic range, and introduced to the new environment there (Blackburn et al. 2011a)—we term these combined stages “translocation”. The transport and introduction stages are often considered together, as here, because we rarely have data on species that have been transported but not introduced: the first evidence that transport has occurred is usually when we observe alien host species already in the wild, especially for host species translocated by accident. Translocated host species tend either to be actively selected by humans (and therefore probably considered beneficial in some way) or, if translocated accidentally, then host species more likely to be chosen at random. The likelihood of translocation is driven by the availability of individuals in the native range (Hulme 2009; Blackburn et al. 2015b). Thus, more widespread and abundant host species are more likely to be translocated, and this is true on average regardless of whether host species are translocated deliberately or accidentally (Blackburn and Duncan 2001; Hulme 2009).

These same criteria will apply to parasite species, just as to their hosts, albeit with the additional complication that most parasites translocated accidentally depend on the translocation of their hosts (Lymbery et al. 2014). Thus, parasite species may be translocated deliberately because they are themselves desirable species, for example, biocontrol agents against (possibly alien) pests (Beirne 1975; Hopper and Roush 1993). Alien parasites should be more likely to be translocated accidentally if they are parasites of desirable (to humans) hosts, or of hosts more likely to be translocated accidentally, or if they have free-living stages that are likely to be translocated accidentally. However, even parasites that do inhabit host species may still fail to be translocated if they are not present in the specific host individuals translocated—termed ‘missing the boat’ (Paterson and Gray 1997). The likelihood that this happens should depend on the prevalence and generalism of the parasite, as parasites with higher prevalence in translocated hosts, or that infect a wide range of hosts, are more likely by chance to make it onto the boat (MacLeod et al. 2010).

What evidence there is for parasites is consistent with the idea that availability does indeed influence the likelihood of translocation. Thus, Ewen et al. (2012) found that alien strains of avian malaria in New Zealand had larger native geographic ranges, and were also found in a broader taxonomic range of native host bird species. Although Ewen et al. (2012) had no data on which strains where actually translocated (those present now may only be a small fraction of these), the patterns are consistent with a positive effect of availability (see also next section).

Parasites that make it on to the ‘boat’ may still fail to arrive at the boat’s destination if their hosts die en route—and as discussed above, the presence of the parasite may increase the likelihood of that happening. We would therefore expect a negative relationship between the probability of successful translocation and parasite virulence, given that hosts of more virulent parasites are less likely to survive the voyage (Strauss et al. 2012; Lymbery et al. 2014). At present, we are unaware of any evidence on the impact of virulence on the likelihood that parasites fail in transit. Nevertheless, there is evidence that hosts do frequently die in transit (e.g. Pipek et al. 2015), and the impact of parasites is one possible cause.

### Establishment and Spread

**Fig. 2:**
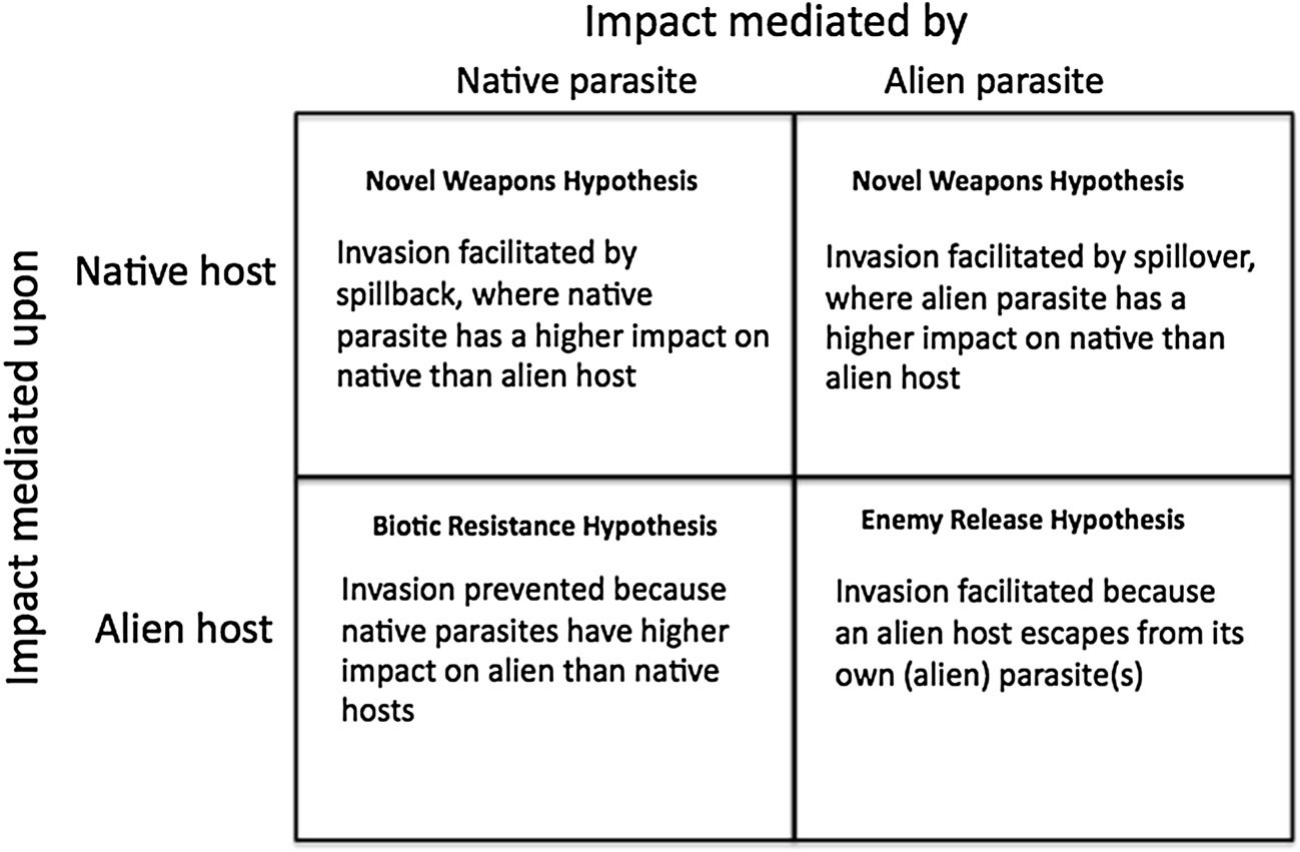
Hypotheses for the impacts of parasites on the potential for invasion by their hosts.

Following translocation, a species must establish a viable population if it is to maintain itself as an alien at the new location (Blackburn et al. 2011a). New environments pose potentially significant challenges to alien population establishment, and only a fraction of translocated species succeed (the much-discussed “Tens Rule” suggests a typical range of 5–20%; see Jeschke 2014). These challenges will be compounded for alien parasites because they are dependent first on the establishment success of their hosts. Parasites that catch the boat and survive the voyage will not become aliens if their hosts fail to establish following arrival; this is termed ‘sinking with the boat’ (MacLeod et al. 2010). The presence of the parasite may once again affect the likelihood of that happening, although this time the effect may be positive or negative (Figure 2).

The most robust and consistent determinant of the success of alien host species is propagule pressure (see above). A larger host population size is also likely to increase a parasite’s establishment success, by increasing the likelihoods that the parasite has been transported, that some infected hosts have survived the journey, and that new hosts are available to ensure transmission in the new environment (MacLeod et al. 2010).

MacLeod et al. (2010) assessed the importance of different processes in determining whether chewing lice successfully established along with their hosts: bird species introduced from Europe to New Zealand. They found that approximately two-thirds of the species of feather lice present on bird species in their native ranges were absent from these hosts in New Zealand. MacLeod et al. (2010) then used simulations to assess the likelihood that louse species would have been lost at different invasion stages, on the basis of data on the composition and prevalence of louse assemblages in the native and alien ranges, and on the numbers of individuals of different bird species introduced. Their analysis showed that few louse species (in the range of 8–20% of those lost) were likely to have missed the boat, because sufficiently high numbers of birds were introduced that most lice species would have been translocated too. Rather, most louse species lost (around half) were most likely to be absent because their hosts failed to establish (they ‘sank with the boat’). Host bird populations were more likely to fail to establish in New Zealand if they comprised lower numbers of individuals (Veltman et al. 1996; Duncan 1997; Green 1997; Cassey 2001; Duncan et al. 2006). Clearly, the situation is likely to vary on a case-by-case basis, depending on the numbers of hosts transported and the prevalence (and any harmful effects) of parasites. It is also likely to depend on the parasites’ host ranges, as more generalist parasites presumably are more likely to be present on at least one host that establishes.

The second most common cause of failure in the louse species studied by MacLeod et al. (2010) was what they termed being ‘lost overboard’: these are parasites that failed to establish alien populations despite being unlikely to have missed the boat, and despite having hosts that successfully established. These species may have died in transit (dealt with above), or failed to establish after translocation. If the latter, then failure may again have been because numbers were against them. As with their hosts, parasites may fail to establish for stochastic reasons if introduced in low numbers (e.g. <5 infected hosts), even in otherwise suitable circumstances (Hatcher et al. 2012). Parasites with single hosts and density-dependent transmission also have a threshold host density below which persistence is unlikely, equivalent to an Allee effect in host populations (Hatcher et al. 2012). Other specific features of the introduction event may also affect success. For example, seasonality (e.g. introduction date) can affect the transmission of parasites by determining the presence of insect vectors (Hatcher et al. 2012).

As well as factors specific to a given introduction event, such as numbers introduced or date, alien species establishment success is influenced by characteristics of the introduction location and of the species introduced (Duncan et al. 2003). The kinds of location-level and species-level effects that influence parasite establishment success are likely to be somewhat different to those for their hosts. The environment for the parasite is the host, and so as long as the parasite’s host establishes, a suitable environment is at least partly guaranteed. Nevertheless, other location-level features can still influence success. Most notably, parasites that are vector-borne or have complex life cycles require the presence of suitable vectors or intermediate hosts, and will not be able to establish in environments lacking them (Prenter et al. 2004; Lymbery et al. 2014). Thus, it was only after the introduction of the alien mosquito *Culex quinquefasciatus* to Hawaii in 1826 that avian malaria could establish in the resident avifauna of the islands (Warner 1968).

A corollary of the requirement for vectors or intermediate hosts is that directly or vertically transmitted parasites are more likely to find the novel environment suitable, assuming that their hosts do (Hatcher and Dunn 2011). Lymbery et al. (2014) found that 64% of co-introduced alien parasites in their literature survey had direct life cycles, but noted that this could be affected by a taxonomic bias in their data towards monogeneans, all of which have direct life cycles. Direct and indirect life cycles were more or less equally represented if monogeneans were excluded. Parasites may also be more likely to establish if they have a broad host range, assuming that this translates into a higher availability of suitable hosts in the new location. Thus, alien strains of avian malaria successfully established in New Zealand have a broader taxonomic range of native host bird species than expected by chance (Ewen et al. 2012). Host range may also interact with life cycle, as vector-borne parasites are more likely to jump hosts (Hatcher et al. 2012). This spillover will be more likely when contact with a novel host is more frequent (Hatcher et al. 2012).

Nevertheless, the presence of novel hosts may actually hamper the establishment of a parasite if the parasite has lower fitness in the new host, as may well be the case when the host and parasite have not co-evolved (Dunn 2009). Most studies of host-parasite interactions consider fitness consequences for the host, but it is the fitness of the parasite that is relevant in the context of parasite invasions. Parasites can establish when their basic reproductive number *R*_0_  > 1: that is, when each primary case of infection results in more than one secondary case (Anderson and May 1982). Transmission into novel hosts in which the parasite cannot complete its life cycle, or for which its virulence is so high that the host dies before it can pass on the infection, can both lower *R*_0_ and cause establishment failure in the parasite (Hatcher et al. 2012). Models of alien bird species establishment also suggest that a high *R*_0_ (for birds, the average number of daughters produced per female over her lifetime) is a key determinant of success (Cassey et al. 2014), implying that demography is likely to matter for both parasites and their hosts (Sol et al. 2012).

Invasive spread by an alien species can be viewed as a continuation of the establishment phase, in which the processes determining establishment are simply played out across a wider environmental arena (Blackburn et al. 2015b). Nevertheless, spread may be facilitated by evolutionary changes in the parasite or host populations following establishment (Hatcher et al. 2012). For example, the evolution of reduced virulence in a novel host in the alien environment may elevate *R*_0_ > 1 for the parasite, and hence promote its spread through the novel host population. Conversely, evolutionary changes that allow host jumps may cause the parasite to act as a novel weapon, promoting its spread via an expansion of its original (alien) host (see above). Selection is most likely to drive evolutionary changes in situations where *R*_0_ is close to (but below) 1 (Holt et al. 2005), as populations with *R*_0_ ≪ 1 will die out too rapidly for selection to influence their trajectory, while populations with *R*_0_ > 1 will grow without the need for evolutionary adaptations.

## Implications

Alien species constitute an enormous experiment in nature that may potentially provide insights into how nature is structured (Blackburn 2008). Parasites have frequently been invoked as drivers of invasions, but have received less attention as passengers, especially when one considers that they may comprise half of all species. Our review suggests that the evidence to date that parasites drive invasions by hosts is weak. There is as yet no really convincing evidence for the ERH, NWH or BRH, in respect to parasites, as determinants of success in alien host establishment or spread. Particular case studies are suggestive—and their consequences in some cases devastating—but not yet informative about general effects. What evidence there is for parasites as aliens suggests that the same kind of factors determine their success as for non-parasites. Thus, availability is likely to be an important determinant of translocation. Establishment and spread are likely to depend on propagule pressure, and on the environment being suitable (in this case, all necessary hosts and vectors are present)—the likelihood of both of these dependencies being favourable will be affected by traits relating to life history and demography. The added complication for the success of parasites as aliens is that often this will depend on the success of their hosts as aliens. This suggests in turn that the average success of alien parasites is likely to be lower than for their hosts—and it would be interesting to revisit the Tens Rule for alien parasites.

It is commonly (though wrongly) assumed that biologists consider all aliens to be undesirable, but sometimes we want species to succeed as aliens. Examples include biocontrol agents and some conservation translocations, notably species undergoing assisted colonisation or being introduced as ecological replacements (Seddon et al. 2014). Protocols for the introduction of biocontrol agents are now strict, and pay close attention to the potential dangers of co-introducing parasites, or of introducing parasites that will have detrimental impacts on non-target organisms (IPPC 2005). Likewise, there are also detailed guidelines for conservation translocations that recognise the need to manage parasite transfer, and that prescribe disease risk assessments (IUCN/SSC 2013). However, these guidelines also stress that it is not possible (or necessarily desirable) to guarantee that translocated organisms are parasite-free (or will have non-target effects), and therefore, our review of parasites in the context of alien invasions may provide useful information for these eventualities.

First, it is likely that, unless steps are specifically taken against them, parasites will be co-introduced, and hence that successfully translocated organisms will carry their parasites. The probability of co-introduction will be increased given that aliens we wish to succeed will typically be introduced in as large numbers as possible to avoid the perils of small population size. However, the likelihood of co-introduction will be lower for rare parasites or for parasites of rare hosts (or biocontrol cultures deriving from small numbers of founders) that may have lost parasites by chance because they passed through a bottleneck.

Second, the likelihood of co-introduction will be lower for highly virulent parasites and for horizontally transmitted parasites relative to those transmitted vertically (Prenter et al. 2004; Lymbery et al. 2014). Therefore, while we would expect co-introduction, the co-introduced parasites are less likely to be damaging to the translocated host. The impact of a parasite may be further reduced if it experiences a reduction in its genetic diversity because co-introduction also involves a bottleneck for its population; this may reduce its potential to evolve in response to the host’s immune defences (Blackburn et al. 2015b).

Third, co-introduction is more likely for parasites with broad host ranges (Ewen et al. 2012), which may increase the likelihood that these can have impacts upon native species. Generalist parasites moved to a destination with naïve hosts are particularly high risk (IUCN/SSC 2013), as illustrated by avian malaria in Hawaii (Warner 1968).

However, fourth, co-introduction of parasites with narrow host ranges does not guarantee that those parasites will not have impacts upon native species. Parasites are more likely to spillover to close phylogenetic relatives (Strauss et al. 2012), at least in animals, while there is some evidence that their hosts are more likely to establish in locations with close phylogenetic relatives (reviewed in Park and Potter 2013). The fact that co-introduced parasites are likely to be those with lower virulence in the normal (alien) host (see above) means that we might expect them to be more damaging, on average, to naïve native hosts. Thus, Lymbery et al. (2014) identified 76 co-introduced parasites in their literature review that had switched to native hosts, of which 16 species had information on relative virulence. Fourteen of these parasites were more virulent in the (new) native than the co-introduced alien host. Furthermore, the alien hosts can act as reservoirs for the co-introduced parasites when they are relatively avirulent in these natural hosts (Lymbery et al. 2014).

Fifth, we would expect that biotic resistance from native parasites would in general matter more than the novel weapons of alien parasites, because the natives should outnumber the aliens. The effects of biotic resistance may be greatly underestimated because they should primarily relate to failed introductions, whereas we can usually only effectively study successful introductions. Nevertheless, the odds are not always on the side of the natives. Alien parasite introductions have been responsible for some of the most serious changes in natural communities in recent decades (e.g. rinderpest, chestnut blight) (Dunn and Hatcher 2015), and such examples make us rightly wary of co-introduction, whatever the odds. Alien parasite impacts may exhibit “pink noise”, where their magnitude is inversely proportional to their frequency (Halley 1996).

Finally, we have been focussing on parasites that might be co-introduced with alien hosts, but similar risks may pertain when the host is a native species. Translocation of individuals for reintroduction or reinforcement (Seddon et al. 2014) may introduce alien parasites if the host has been in a captive breeding facility outside the native range, or alongside species it would not normally encounter (e.g. Walker et al. 2008), or if the individuals come from disjunct populations in other parts of the species’ native range, in these scenarios effectively crossing ecological or geographic boundaries (Bobadilla et al. this special issue). However, even the reintroduction of native parasites may have negative impacts on the native biota, for example, if the abiotic environment has changed significantly in the meantime, if declines in native biodiversity open the way for an increased incidence of parasites in the species that remain (Hatcher et al 2012), or if introduced hosts alter contact rates with resident hosts facilitating increased parasite transmission (Aiello et al. 2014). This is a concern because parasites are species too, and there is no reason why they should not be as deserving of conservation attention as other species (Jørgensen 2015).
